# Addition of Multimodal Immunotherapy to Combination Treatment Strategies for Children with DIPG: A Single Institution Experience

**DOI:** 10.3390/medicines7050029

**Published:** 2020-05-19

**Authors:** Stefaan W. Van Gool, Jennifer Makalowski, Erin R. Bonner, Oliver Feyen, Matthias P. Domogalla, Lothar Prix, Volker Schirrmacher, Javad Nazarian, Wilfried Stuecker

**Affiliations:** 1 Immun-Onkologisches Zentrum Köln, Hohenstaufenring 30-32, 50674 Köln, Germany; makalowski@iozk.de (J.M.); domogalla@iozk.de (M.P.D.); v.schirrmacher@web.de (V.S.); stuecker@iozk.de (W.S.); 2 Center for Genetic Medicine, Children’s National Health System, Washington, DC 20010, USA; ebonner@childrensnational.org; 3 Institute for Biomedical Sciences, The George Washington University School of Medicine and health Sciences, Washington, DC 20052, USA; 4 Zyagnum, Reißstrasse 1, 64319 Pfungstadt, Germany; oliver.feyen@zyagnum.com; 5 Biofocus, Berghäuser Strasse 295, 45659 Recklinghausen, Germany; l.prix@ladr.de; 6 DIPG Research Institute, Universitäts-Kinderspital Zürich; Steinwiesstrasse 75, Ch-8032 Zürich, Switzerland; javad.nazarian@kispi.uzh.ch

**Keywords:** DIPG, multimodal immunotherapy, DC vaccination, Newcastle disease virus, hyperthermia, PanTum Detect test, Th1 shift

## Abstract

**Background**: The prognosis of children with diffuse intrinsic pontine glioma (DIPG) remains dismal despite radio- and chemotherapy or molecular-targeted therapy. Immunotherapy is a powerful and promising approach for improving the overall survival (OS) of children with DIPG. **Methods**: A retrospective analysis for feasibility, immune responsiveness, and OS was performed on 41 children treated in compassionate use with multimodal therapy consisting of Newcastle disease virus, hyperthermia, and autologous dendritic cell vaccines as part of an individualized combinatorial treatment approach for DIPG patients. **Results**: Patients were treated at diagnosis (n = 28) or at the time of progression (n = 13). In the case of 16 patients, histone H3K27M mutation was confirmed by analysis of biopsy (n = 9) or liquid biopsy (n = 9) specimens. PDL1 mRNA expression was detected in circulating tumor cells of ten patients at diagnosis. Multimodal immunotherapy was feasible as scheduled, until progression, in all patients without major toxicity. When immunotherapy was part of primary treatment, median PFS and OS were 8.4 m and 14.4 m from the time of diagnosis, respectively, with a 2-year OS of 10.7%. When immunotherapy was given at the time of progression, median PFS and OS were 6.5 m and 9.1 m, respectively. A longer OS was associated with a Th1 shift and rise in PanTum Detect test scores. **Conclusions**: Multimodal immunotherapy is feasible without major toxicity, and warrants further investigation as part of a combinatorial treatment approach for children diagnosed with DIPG.

## 1. Introduction

Diffuse intrinsic pontine glioma (DIPG) is a rare brainstem tumor that typically occurs in children. The overall incidence rate of all primary brain tumors ranges between 3–6 per 100,000 children and adolescents between 0 and 19 years of age [1,2]. About 10–15% of these tumors are located in the brain stem [2,3] and 75–80% of pediatric brainstem tumors are DIPG [3], making its incidence less than one per 100,000 children each year. DIPG is diagnosed by assessment of clinical symptoms derived from the pyramidal tract, the cerebellum, and the cranial nerves, together with the typical imaging on MRI, which manifests as a T1-weighted hypointensity,T2-weighted hyperintensity involving >50% of the pons, with occasional ring contrast enhancement [4]. Most, but not all, of these lesions are driven by histone mutations [5], classifying them as diffuse midline gliomas (DMG), according to the current WHO brain tumor classification [6]. About 85% of DIPGs harbor histone mutations [7].

There is no known cure for DIPG [7] and the 2-year overall survival (OS) remains below 10%. Insufficient data in the literature preclude analysis of the 5-year OS of this disease [8]. The poor prognosis of DIPG is in contrast to the more favorable prognosis of pediatric glioblastoma multiforme (GBM), which results in a 5-year OS of about 20% in cases involving favorable clinical risk factors (e.g., age, location, extent of resection) [9]. In contrast, DIPG is inoperable, and radiotherapy is generally accepted as the standard-of-care treatment, reducing temporarily acute symptoms [10,11,12,13]. Changing radiotherapy modalities, or the addition of (neo)adjuvant chemotherapy or other drugs, has not changed the OS over the past 30 years [14]. Re-irradiation is possible [15]. A breakthrough came when biopsy was developed as a safe and feasible procedure [16,17]. Knowledge on the molecular biology of the tumor has resulted in the development of molecular-driven treatment strategies [18,19]. The worldwide networks for biology data and the DIPG registry for clinical and radiological data together form the basis for future development in the field [20,21]. Apart from radiotherapy as a single treatment modality or clinical trials combining radiotherapy with classic chemotherapy [22], innovative treatment modalities have emerged such as local treatments via convection-enhanced delivery [23,24,25]. 

Immunotherapy and biologic treatments for DIPG are emerging fields of research. A Clinicaltrials.gov search (September 2019) for “DIPG/Immunotherapy” yielded six trials studying the role of vaccines including two trials with dendritic cell (DC) vaccines (NCT03396575, phase 1, recruiting; NCT02840123, phase 1, active, not recruiting), two trials focused on immunomodulation with Indoximod (NCT04049669, phase 2, not yet recruiting; NCT02502708, phase 1, recruiting), one trial with tumor-initiating-cells (NCT01400672, phase 1, suspended), and one with the H3.3K27M peptide vaccine (NCT02960230, phase 1, active, not recruiting). One trial was registered using the oncolytic virus Adenovirus DNX-2401 (NCT03178032, phase 1, recruiting). Each of these studies included a limited number of patients, and focused on feasibility, toxicity, or preliminary efficacy. Treatments typically included radiotherapy. Data from these trials would give important, but limited, information on the research question. 

In addition, individualized combination treatment strategies outside of clinical trials are a well-known phenomenon, not only in the scientific community but also in patient communities. Particularly for DIPG, treatment approaches outside of clinical trials are common [26]. It is of utmost importance for both the scientific and patient community that the results from these individualized treatment approaches are analyzed and reported. We therefore aimed to present the data of a retrospective analysis of 41 children with DIPG consecutively treated with multimodal immunotherapy in compassionate use at the Immun-Onkologisches Zentrum Köln (IOZK), between October 2011 and February 2018.

## 2. Materials and Methods 

The database consisting of all patients who had contact with the IOZK was fixed at 15 July 2018. A search for DIPG as the primary diagnosis yielded 142 records of patients from at least 33 countries (data not available for 16 patients). Forty-one of these patients (29%) were actively treated with immunotherapy at the IOZK as an individualized treatment approach in compassionate use (“individueller Heilversuch”) between October 2011 and February 2018. Thirty-six patients (88%) began treatment between 2016–2017. The last OS analysis was performed at the end of September 2019.

All patients started immunotherapy with a blood investigation focused on the functional status of the immune system. The immunotherapy consisted of vaccination cycles and/or immunogenic cell death (ICD) therapy. Full vaccination cycles consisted of five consecutive days of treatment with intravenous injection of Newcastle disease virus (NDV) in combination with local modulated electrohyperthermia (mEHT) via the Oncothermia EHY-2000 device (Oncotherm GmbH, Troisdorf, Germany) for 40 min at an intensity of 40 W. During mEHT, 0.9% NaCl infusion was administered. On the eighth day, a sixth session of NDV/mEHT was administered. Autologous mature DCs were loaded with NDV/mEHT-induced serum-derived antigenic extracellular microvesicles and apoptotic bodies. DCs were injected intradermally in the upper third of the arm. This personalized vaccine, approved as an advanced therapy medicinal product since May 2015 and registered as IO-VAC^®^, was prepared as described [27]. The therapy consisted of two consecutive vaccination cycles with an interval of three weeks between each cycle. ICD therapy consisted of three days of NDV/mEHT. ICD therapy was incorporated at days 8, 9, and 10 in conjunction with 5/28 days oral TMZ. Following the maintenance chemotherapy, vaccination cycles were started following a similar schedule as described for adults with GBM [27]. ICD therapy was also given as maintenance immunotherapy after two full vaccination cycles for all patients who reached that stage. 

Plasma circulating tumor DNA (ctDNA) analysis was used to screen for H3.3K27M mutation in 21 patients, as described previously [28,29]. Briefly, cell free DNA was isolated from 1 mL of plasma, and digital droplet PCR (ddPCR) was performed as per [29] to detect, and quantify the abundance of the *H3F3A* wildtype and K27M mutant alleles. 

Patients were monitored during treatment at three levels. Routine cell numbers and immune functional tests were determined in the clinical laboratory (www.synlab.com). The percentage of IL-4 and IFN-g expression within CD4+ T cells was determined using FACS. Circulating tumor cells (CTC) were isolated and the mRNA expression level of PDL1 was analyzed by Biofocus (www.biofocus.de). The PanTum Detect tests were performed at IOZK using the Epitope Detection In Monocyte (EDIM) technology as described [30,31,32,33]. 

## 3. Results

### 3.1. Patient Characteristics

**Figure 1:**
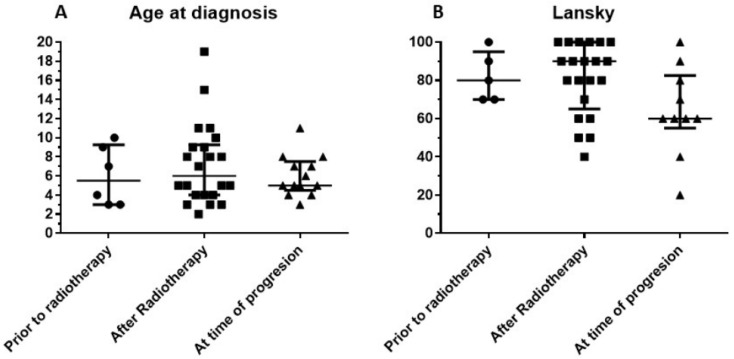
Characteristics of retrospective cohort consisting of 41 children with diffuse intrinsic pontine glioma (DIPG). Patients included in this retrospective cohort were stratified into three groups based on the treatment modalities they received in addition to immunotherapy. Patients in Group 1 received immunotherapy prior to radiation therapy, while patients in Group 2 received radiation therapy before beginning immunotherapy as their first line of treatment. Patients in Group 3 received immunotherapy at the time of progressive disease, following their first line of treatment. (**A**) Age distribution (median and interquartile range). (**B**) Lansky score at admission for immunotherapy (median and interquartile range).

Forty-one children (n = 10 male, 31 female) from 16 countries were effectively treated with multimodal immunotherapy at the IOZK, following an in-depth explanation of the treatment strategy and written informed consent from the patient or patient’s guardian. Our retrospective analysis focused on this group of patients. An additional 101 children with DIPG were registered in the database, but did not follow treatment for various reasons. The treated children were subdivided into three groups: Group 1, children receiving immunotherapy before radio- and chemotherapy (n = 6); Group 2, children receiving immunotherapy in conjunction with the first line of treatment provided by the local oncology center (n = 22 total; radiotherapy only = 13, radiotherapy and chemotherapy = 9); and Group 3, children treated with immunotherapy upon disease progression following the first line of standard treatment, which consisted of radiotherapy for all patients, and a combination of radio/chemotherapy in the case of nine patients (n = 13 total). The median age at diagnosis for all children was five years, with a range from two to 19 years (Figure 1A). The age range did not differ between the three patient groups (Kruskal–Wallis test). The median Lansky Playing Scores (LPS) of the three patients groups were 80, 90, and 60, respectively, indicating no significant difference between the groups (Kruskal–Wallis test), although the minimum LPS were 70, 60, and 20, respectively (Figure 1B). 

All children received the diagnosis of DIPG based on MRI diagnostics in the local treating oncology center and were counseled accordingly. A retrospective central radiology review was not organized. Data on the length of symptoms prior to diagnosis were not systematically captured. Histone mutations were molecularly confirmed by tissue biopsy in nine patients (19.5%) (n = 1/6 patients from Group 1, 5/22 from Group 2, and 3/13 from Group 3). ddPCR analysis of plasma ctDNA indicated that 9/21 patients tested were positive for H3.3K27M mutation [34,35]. Plasma ctDNA mutation detection was in accordance with histone mutation status confirmed by tissue biopsy in 2/3 patients. Hence, molecular support for the DIPG diagnosis was present in 16/41 patients (n = 1/6 patients from Group 1, 12/22 from Group 2, and 3/13 from Group 3). 

**Figure 2:**
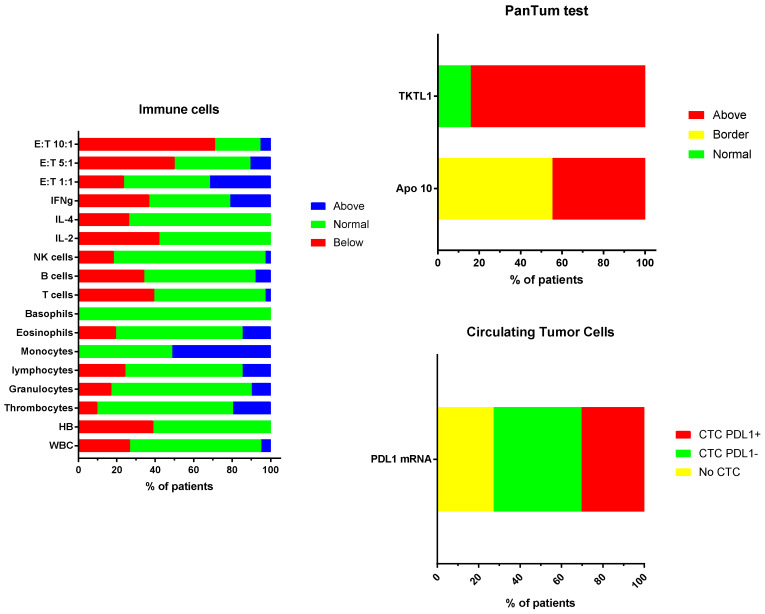
(**A**) Proportion of patients deviating from the normal reference values: White blood cell count, hemoglobin, thrombocyte count; relative values for white blood cell subpopulations as percentage (granulocytes, lymphocytes, monocytes, eosinophils, basophils); absolute values for lymphocyte subpopulations (T cells, B cells, NK cells); percentage cytokine producing CD4+ T cells (IL-2; IL-4, IFN-g) and cytotoxic NK cell function at three effector target ratios (E:T 1:1, 5:1, 10:1). (**B**) Proportion of patients showing levels (normal, border, above the reference values) of Apo10 and TKTL1 within the CD14+CD16+ monocytes. (**C**) Proportion of patients without (no CTC) or with CTCs, and with negative (CTC PDL1-) or positive (CTC PDL1+) PDL1 mRNA expression.

An immune diagnostic procedure was performed on patients prior to the start of immunotherapy (Figure 2). Three different categories of tests were performed. (1) The number and functions of T cells, B cells, and NK cells were compared to the reference values of the laboratory. The relatively high proportion of patients falling below the lower reference limit reflects their first line of treatment with chemotherapy (administered to three patients in Group 1) and/or radiotherapy (administered to all patients in Group 2). The three children who did not receive chemotherapy or radiotherapy from Group 1 had also at least one variable below the reference limit for cytokine production. These children did not receive steroids. Patients from Group 3 were no more affected than patients from Groups 1 or 2. (2) A PanTum Detect test was performed in all children at diagnosis. The PanTum Detect test is a novel screening test based on two general markers in cancer, Apo10 and TKTL1, which can be detected with intracellular staining and FACS analysis in CD14+CD16+ gated circulating monocytes using EDIM technology [30,31,32,33]. The Apo10 protein epitope marks tumor cells with abnormal apoptosis and proliferation. The transketolase-like protein 1 (TKTL1) represents the enzymatic basis for anaerobic glucose metabolism even in the presence of oxygen, which is concomitant with a more malignant phenotype due to invasive growth/metastasis and resistance to radical and apoptosis-inducing therapies. Interestingly, at least one PanTum Detect test score was in the pathologic range in all patients. Only four patients, all belonging to Group 2, had a borderline Apo10 value with a normal value for TKTL1. (3) Although plasma for ctDNA analysis was not systematically sampled at diagnosis, circulating tumor cell (CTC) detection was performed in almost all children. The latter test isolates CTCs derived from brain tumors as these cells are larger than circulating blood cells, remain on top of a filter, and harbor oncogenic mRNA expression profiles (e.g., elevated expression of telomerase, ERBB2, C-kit, and EGFR, relative to the expression of housekeeping genes). CTCs were detected in 2/5 patients from Group 1, 17/20 patients from Group 2, and 5/8 patients from Group 3. Increased PDL1 mRNA expression in CTCs was detected in one, seven, and two patients from Groups 1 to 3, respectively. In five patients with positive CTC detection, PDL1 mRNA expression was not elevated compared to the housekeeping genes. In 3/9 patients tested, no CTCs were detected in the blood. In one biopsied patient from Group 3, no CTC data were available. Together, the immune function variables, the PanTum Detect test variables, and the evidence of PDL1 mRNA expression in CTCs all indirectly provide information on the tumor microenvironment including the interaction between the tumor and the host’s immune system. The results of these tests can inform and refine personalized immunotherapy. 

### 3.2. Treatment Data

Before the start of immunotherapy, three patients in Group 1 received chemotherapy prior to radiotherapy, according to the Polish standard of care. One of these patients received a complex cocktail of different repurposing and complementary drugs (Agomelatine, minocycline, valproic acid, curcumin, Boswelia, scorpion venum extract, CBD), and another patient received photodynamic treatment (PDT, www.webermedical.com). This latter child and one additional patient were further treated with PDT during immunotherapy. Patients in Group 2 were all treated with radiotherapy prior to immunotherapy. Nine patients also received chemotherapy, five of whom continued Temozolomide (TMZ) maintenance chemotherapy together with immunogenic cell death (ICD) treatment until the first progression, after which full dendritic cell (DC) vaccination cycles were initiated. One patient was treated respectively with CED treatment, oral panobinostat, gallium maltonate, and Bevacizumab as part of the first line of treatment prior to immunotherapy. During immunotherapy, one patient was also treated with CED, PDT, and gallium maltonate, respectively. As a result, 14/22 patients from Group 2 received multimodal immunotherapy following radiotherapy without any concomitant treatment modality. Two of the 13 children in Group 3 combined multimodal immunotherapy with gallium maltonate treatment. The use of complementary drugs or diet was not systematically reviewed. 

**Figure 3:**
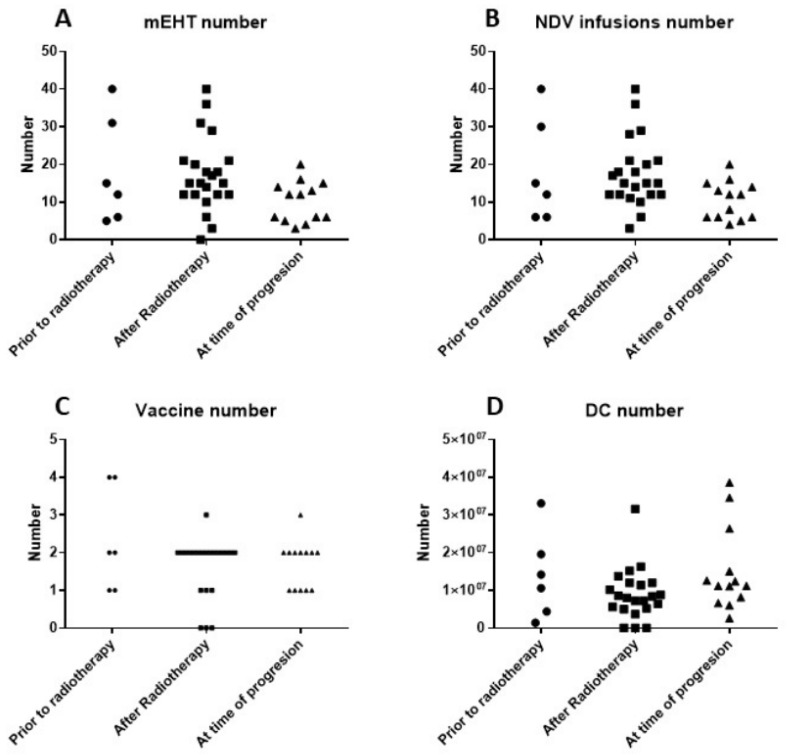
(**A**) Number of sessions of modulated electrohyperthermia (mEHT); (**B**) number of injections of Newcastle disease virus (NDV); (**C**) number of dendritic cell (DC) vaccines; and (**D**) number of DCs injected for the three patient groups.

The technical details of the multimodal immunotherapy are shown in Figure 3. ICD treatment consisted of the combination of intravenous NDV administration together with mEHT. Each DC vaccination cycle consisted of six NDV/mEHT treatments combined with intradermal injection of autologous mature DCs loaded with NDV/mEHT-induced serum-derived antigenic extracellular microvesicles and apoptotic bodies from the patient’s tumor. There was no significant difference in the numbers of DCs, vaccinations, hyperthermia sessions, or NDV administrations between the three groups of patients (Kruskal–Wallis test).

### 3.3. Clinical Evolution

All treatments were administered in an ambulatory setting. Twenty-four children received a central venous access device placed prior to the start of immunotherapy, while 15 children received the immunotherapy without central venous access. Two children received a central venous access during immunotherapy. One child with fast progressive DIPG and LPS of 40 received a central venous access device, aimed at providing general support including a short hospitalization. This patient discontinued immunotherapy after one week. Treatment-induced side effects were not systematically screened using questionnaires. At each patient contact, clinical signs were discussed. Most symptomatology was attributed to the DIPG itself and/or other antitumoral treatments given. Nevertheless, low grade complaints of fever (n = 1), dizziness (n = 2), neuralgia (n = 1), and headache (n = 1), all likely related to immunotherapy, and ascites in the context of a ventriculo-peritoneal drainage (n = 1) were registered in the database. 

**Figure 4:**
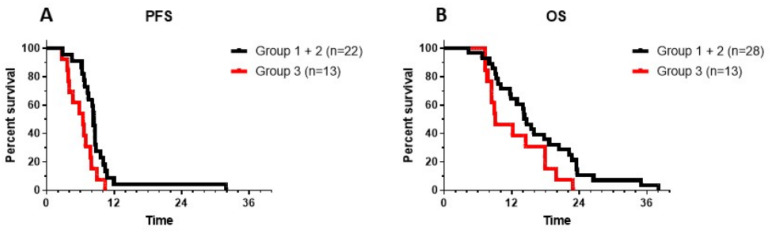
Progression-free survival (PFS) (**A**) and overall survival (OS) (**B**) are shown for patients from Groups 1 and 2 (black line: patients receiving immunotherapy as the first line of treatment either before (Group 1) or after (Group 2) radiotherapy) and from Group 3 (red line: patients receiving immunotherapy at the time of progressive disease, following the first line of treatment). Log-rank test for PFS showed a *p* value of 0.014. Log-rank test for OS showed a *p* value of 0.057.

MRI was performed at the local oncology center. Progressive disease can be difficult to discern in the context of DIPG, and, reference radiology was not systematically performed in all patients, further complicating the identification of disease progression. Moreover, the contribution of immunotherapy made the interpretation of MRI findings more difficult. IOZK provided all information when requested for the assessments at the local hospitals. For the analysis in this retrospective study, PFS was defined as the moment when a new treatment strategy was implemented by the local oncology center. Data were available for 22 patients from the 28 first-line DIPG patients belonging to Group 1 (immunotherapy before radiotherapy) and Group 2 (immunotherapy after radiotherapy). The median PFS was 8.4 m (Figure 4A). The six-month PFS was 90.9% (CI95%: +6.7, −22.6). We recognized that our patient group was highly biased with children still being able to travel, and with parents putting maximal effort and resources for treatment. A similar profile, however, was present in the 13 children from Group 3 who came for immunotherapy at the time of progressive disease. In these children, we calculated PFS as the time between diagnosis and the date of a second event, hence first-line treatment without immunotherapy. For these 13 children, the median PFS was 6.5 m, and the 6-month PFS was 53.8% (CI95%: +22.1, −29.0), which was significantly (Log-rank test: *p* = 0.013) less than that of the patients who received immunotherapy as part of their first-line treatment. 

It is generally accepted that OS is the ultimate outcome to be considered in patients with DIPG. A similar approach was therefore performed to assess the OS of patients from each group. Data from all children were available, and all had an event. The median OS of the Groups 1 and 2 patients combined was 14.4 m, with a 1-year OS of 64.3% (CI95%: +14.6, −20.5), and a 2-year OS of 10.7% (CI95%: +14.3, −8.0) (Figure 4B). The longest OS of patients in Groups 1 and 2 was 38 m (Figure 4B). There was no significant difference between Group 1 (median OS: 16.9 m; 2-year OS: 16.6%, CI95%: +35, −15.9) and Group 2 (median OS: 14.4 m; 2-year OS: 9%, CI95%: +16, −7.5). The OS for the patients in Group 3, calculated from the time of initial diagnosis, was 9.1 m, with the longest OS of 22.9 m. There was a trend toward a longer OS when patients were treated with immunotherapy as part of first-line treatment (*p* = 0.057). 

Two patients from Group 3 with progressive disease were treated prior to 2015. One patient from Group 1 began treatment in September 2015. One patient from Group 2 began treatment in February 2018. All other patients were treated between 2016–2017. Thus, the general policy for rescue treatments was quite homogeneous. A total of 8/28 patients from Groups 1 and 2 received re-irradiation upon progression. One of these eight patients received CED therapy in London, and subsequently received intra-arterial chemotherapy in Monterrey (idoimexico.com). One other patient proceeded with antineoplaston treatment followed by intra-arterial chemotherapy in Monterrey. We are not aware whether chemotherapy rescue protocols were initiated. Most patients went to palliative treatments.

### 3.4. Laboratory Data Monitoring

**Figure 5:**
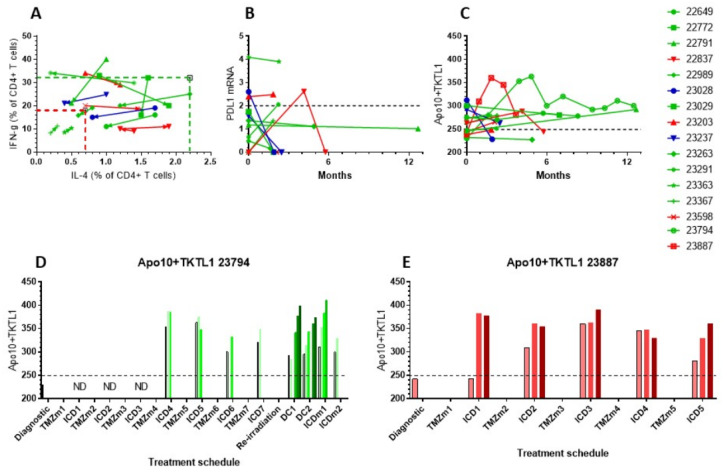
Evolution of immune variables in blood was followed in 14 patients. Patients treated with immunotherapy as part of their primary treatment with an OS longer than the median OS are shown in green, and patients with an OS shorter than the median OS are shown in red. Marked in blue are two patients treated with immunotherapy at the time of progressive disease following the first line of treatment. (**A**) Percentage IL-4 and IFN-g producing CD4+ T cells measured at different time points. The arrow indicates the evolution. (**B**) Longitudinal PDL1 mRNA expression in CTCs over the course of 12 months. The reference cut-off was 2 (dashed line). (**C**) The evolution of the sum of Apo10 and TKTL1 over 12 months. The maximum cut-off was 249 (dashed line). (**D**,**E**) Individual data of Apo10 + TKTL1 scores in patients 23794 and 23887. Each treatment block is indicated. During each immunotherapeutic intervention, Apo10 + TKTL1 scores were measured before the start of, and at each consecutive day during treatment.

Data on the evolution of the above described values over time were available for 14 patients with longer follow up (one patient from Group 1, 11 patients from Group 2, and two patients from Group 3). Data on the PanTum Detect test scores were available for two further patients from Group 2 (23794 and 23887). Ten patients from Groups 1 and 2 had an OS that was longer than the median OS, while four patients had an OS that was shorter than the median OS. Data on the shift in Th1/Th2 balance are shown in Figure 5A. Although mostly within the normal reference range, 5/9 patients with an OS longer than the median shifted toward Th1 upon immunotherapy, and 4/9 toward Th2. The three patients with a shorter OS all shifted toward Th2 upon immunotherapy. One patient (22837) first shifted to Th1, but then clearly to Th2. The median OS of the patients that shifted to Th1 was 23.5 months, whereas the median OS of the patients that shifted to Th2 was 17.7 m (not significant). The two patients from Group 3 shifted toward Th1 immediately following their two vaccination cycles. 

Available mRNA values for PDL1 in CTCs (Figure 5B) did not change significantly over time as compared to the immunodiagnostic test. Values for the sum of Apo10 and TKTL1 scores were also followed over months in this subset of patients (Figure 5C). The values reflect the content of tumor-derived Apo10 and TKTL1 within CD14+CD16+ monocytes in peripheral blood. The levels of these values are influenced by the volume of tumor cell damage as well as tumor cell death in response to treatment. The two patients treated at the time of relapse had a reduction in Apo10 and TKTL1 scores over time. Additionally, two patients from Groups 1 and 2 with shorter than median OS had decreasing values, while patients from Groups 1 and 2 who showed longer than median OS had mostly stable or increasing values, reflecting persistent uptake of Apo10 and TKTL1 from dying tumor cells. The more detailed curves of patients 23794 and 23387 showed an initial increase followed by a stable and decreasing curve, respectively (Figure 5C). Of note, these two children were treated according to the German standard of care with radiochemotherapy and up to 12 maintenance TMZ cycles, to which ICD treatments were added, similar to the schedule published previously for adults with GBM [27]. At the immune diagnostic blood sampling, both patients had a low content of Apo10 and TKTL1 in the CD14+CD16+ monocytes, though both were treated only with radiochemotherapy. Both patients showed an increase in their PanTum Detect test scores and a subsequent decrease, potentially reflecting the effect of transient radiochemotherapy and maintenance chemotherapy plus ICD therapy. Patient 23794 received seven TMZ cycles with ICD treatment, experienced disease progression, and was then re-irradiated and received two DC vaccination cycles and further maintenance ICD treatments. Patient 23887 received five TMZ maintenance cycles with ICD treatment, experienced disease progression, and finished immunotherapy, but then received re-irradiation and anti-GD2 antibody. 

Focusing on the evolution of the PanTum Detect test results as a marker of response to ICD treatment [36], we began daily PanTum Detect test measurements during treatment. Data from two patients (23794 and 23387) were available. Figure 5D,E show the daily evolution of the available PanTum Detect test results during immunotherapy at different treatment episodes. The retrospectively sampled dataset was incomplete, however, one can appreciate the marked increase in the PanTum Detect test results in Patient 23887 upon the first 3-day ICD treatment (Figure 5E). The ICD treatment did not induce a further increase in the PanTum Detect test scores on a day-by-day basis when the starting value of the treatment block was increased, but the increase in the PanTum Detect test scores became clear again when the starting value of the treatment block was lowered. In Patient 23794, at the time of DC vaccinations and the 5-day ICD treatment, an apparent day-by-day increase was again observed upon injection with NDV and treatment with mEHT (Figure 5D). Together, the data suggest that the values of the PanTum Detect test scores evolve over time, and that this evolution is likely to be influenced by both the response to the standard antitumor treatment (transient increase and decrease over months) and the response to ICD treatment (rapid increase day-by-day).

## 4. Discussion

In this study, we summarized the data obtained from a retrospective analysis of a cohort of children diagnosed with DIPG who received multimodal immunotherapy as a primary treatment, or at the time of progressive disease, at the IOZK mainly between 2016–2017.These children were treated using an individualized approach. As such, the data should be taken with great caution, and no firm conclusions can be drawn. The authors, however, do believe that the reporting of such data is of high value to the field of DIPG research, and also holds value for the community of patients and their families. It is critical to provide comprehensive data gathered from retrospective analyses, in order to aid in (1) the counseling and guidance of future patients on the basis of the analyzed data, and (2) the development of new innovative clinical trials.

Immunotherapy and molecular biology-based treatments are emerging in the field of DIPG. H3K27M is recognized as a tumor-specific antigen [37,38], and vaccination strategies using the long-peptide are under consideration. Immune responses have been generated using autologous DCs loaded with lysate from DIPG cell lines [39]. Moreover, DC vaccination technology has been shown to be feasible and safe. The tumor microenvironment remains a major obstacle in immunotherapy approaches [40], and oncolytic virus therapy can play a major role in modulating the tumor microenvironment [41,42]. NDV was shown in vitro to reduce the viability of DIPG cell lines (Carolien Koks, unpublished data). Infiltration of tumor-reacting T cells within a DIPG microenvironment was demonstrated in animal models upon treatment with the Delta-24-RGD oncolytic virus [43,44]. A concern in the field of oncolytic virus therapy is the antiviral immunity of the patient. However, anti-viral immunity in an animal model using NDV as the oncolytic virus has been shown to potentiate its immunotherapeutic efficacy [45]. mEHT is a method to treat cancer by inducing heat stress, which selectively targets tumor cells due to their altered metabolic dependencies relative to healthy cells, resulting in different conductivity of electromagnetic waves [46]. mEHT is known to induce immunogenic cell death [47,48,49,50], and has been previously utilized in the treatment of brain tumors [51]. Overall, there are sufficient data as well as experience at the IOZK to support multimodal immunotherapy for these children as an individualized treatment approach.

While the patient group is small, the collation of retrospective data from 36 patients with DIPG over a period of two years in one institution remains remarkable. The drive for this retrospective study was mainly the interest both among clinicians and parents of children with DIPG, as evidenced by social media. The authors realize that this is a highly biased patient group at multiple levels including the medical condition and the attitude of the parents. However, when considering the age distribution and LPS distribution of this cohort, the patient group reflects the typical DIPG patient profile. The LPS was very low in some patients from Group 3, presenting with progressive disease. Whereas all patients had typical clinical symptoms and MRI findings to substantiate the diagnosis of DIPG, the diagnosis was further supported in 16/41 patients (39%) by molecular analysis via tissue or plasma liquid biopsy, facilitating the identification of diagnostic histone H3K27M mutations in a subset of patients. Nonetheless, MR imaging was the key factor in DIPG diagnosis. The available data did not allow an appropriate matching analysis between tissue biopsy and liquid biopsy. 

All patients were counseled and treated for DIPG by the oncologic center. Thirteen children from Group 2 were treated with radiotherapy as the first line treatment alone, while in total, three children from Group 1 (Polish protocol, though meant to be followed by radiotherapy) and nine children from Group 2 were treated with radiotherapy and chemotherapy. This distribution also reflects the current reality for DIPG treatment practices [26]: 52% of our patients with first diagnosis were treated by the oncologic center with radiotherapy alone. 

A surprisingly high proportion of patients exhibited immune variables below the reference values at the time of presentation for multimodal immunotherapy. Both the disease itself and the treatments given have a clear effect on immune potency. Even the three children who were not treated with steroids or other prior treatments (radio- or chemotherapy) had levels below the reference limit for at least one functional immune variable (cytokine production, NK killing function). This is a notable finding, as the DIPG tumor microenvironment has been recently characterized as neither highly immunosuppressive nor inflammatory [40]. Furthermore, approximately half of the patients with newly diagnosed DIPG were treated with only radiotherapy prior to blood sampling. However, radiotherapy given only locally can still have a systemic effect on the immune system [52]. The addition of Temozolomide during and after radiotherapy further affects immune function. Nevertheless, a strategy to combine maintenance Temozolomide with immunogenic cell death treatment and subsequent DC vaccination after maintenance chemotherapy has already been published for adults with GBM [27]. Together, the data point to a weakened immune status of the patient at the time of DIPG diagnosis, which is worsened by any further medical intervention. 

Another remarkable finding is the presence of CTCs in the blood at the time of the immunodiagnostic blood sampling prior to immunotherapy. This test, provided by LADR Biofocus, detects CTCs based on filtration techniques for the enrichment of cancer cells from the blood [53,54,55]. Molecular detection is subsequently performed by quantitative real-time PCR to measure the mRNA expression of Telomerase, ERBB2, C-kit, and EGFR in comparison to GADPH mRNA expression. These general markers for brain tumors are used for tumor cell detection, as specific H3K27M mRNA markers are not currently available. CTCs were detected in 73% of patients, a detection rate higher than the detection rate for brain tumors (60%) mentioned by the company. Although there is emerging knowledge on CTCs in patients with GBM [56,57], no specific literature is available for DIPG. Nevertheless, this finding might lead to more systematic detection in these patients, as the presence of CTCs in peripheral blood may reflect changing tumor biology and treatment resistance. Importantly, 42% of CTC-positive samples showed elevated mRNA expression for PDL1 relative to the housekeeping gene mRNA. PDL1 positivity in these CTCs might contribute to immune resistance, similar to the mechanism published for PDL1-expressing GBM-derived extracellular vesicles [58]. Correlations between the expression of PDL1 mRNA in CTCs and data on the tumor–host interaction derived from pathology investigations on biopsies is not currently available, but warrants further study.

All patients were treated with an individualized treatment approach in compassionate use (“individueller Heilversuch”) with the goal of prolonging individual survival and maintaining quality of life. The OS is the most important read-out of any clinical approach for DIPG patients, but remains very poor. All previous attempts to date to improve OS have failed [10,11,12]. The survival prediction model published in 2015 can be considered as actual [13]. In this model, the age, duration of symptoms, and use of chemotherapy are linked to improved OS, whereas ring contrast enhancement on MRI at diagnosis is an unfavorable predictor of OS [13]. In the presented retrospective series, we did not have all the data available to calculate the individual DIPG risk score for each patient. However, only one child from Group 2 was younger than three years of age at diagnosis, and 14/22 (64%) patients did not receive chemotherapy; these two important factors influenced the DIPG risk score for most patients with “+7” in the absence of “−4”. Accordingly, one can estimate that most patients belong to the intermediate and high risk groups. Although the patient cohort in this study remains biased in many respects, and no definite conclusions can be drawn from this retrospective analysis, the observed 14.4 month median OS for Group 2 exceeded the published median OS of 9.7 months for intermediate risk patients and seven months for high risk patients, respectively, in the published survival prediction model [13]. All patients from Group 3 were older than three years, and only four patients (31%) did not receive chemotherapy. The median OS of 9.1 months from diagnosis in this group reflects the published data [13]. These patients represent an equally biased patient group as those from Groups 1 and 2. Therefore, one can conclude that the introduction of immunotherapy as a first line treatment may provide potential benefit, compared to the introduction of immunotherapy at the time of progressive disease. This finding is further supported by the data showing that the estimated PFS shifted by about two months upon introduction of immunotherapy as first line treatment. Immunotherapy at an early stage of disease is generally accepted to be more effective than at a later stage of disease [59]. As most patients did not undergo a tumor biopsy, and hence no tumor DNA was available to determine mutation status or other molecular biology characteristics, we could not further compare the OS data in this study with data published on patient groups defined by the molecular biology of the tumor [60]. 

We were able to measure cytokine production during treatment in several patients. For all monitoring data, patients from Groups 1 and 2 were divided into (a) those living longer or shorter than the median OS, and (b) patients treated at relapse. The authors realize that this is a rough descriptive analysis. A total of 67% of patients with longer OS had an immune response shift toward Th1, while all patients with shorter OS shifted toward Th2. A Th1 shift linked to a better OS upon immunotherapy has also been observed in patients with GBM treated with immunotherapy [27]. In GBM, the profile of the myeloid cells and microglia within the tumor microenvironment contributes to the Th1/Th2 balance, and hence the Th2 shift upon treatment might reflect a more immunosuppressive tumor microenvironment [61]. Similarly, the Th1/Th2 balance has been linked to PDL1/PD2 axis activity [62]. However, the tumor–host mechanisms observed in GBM cannot be transferred to the DIPG tumor microenvironment [40]. Furthermore, the peripheral blood immune status does not necessarily reflect the intra-tumor status. We were not able to detect links between oncologic, immune, or treatment characteristics predicting the shift direction. 

During the years in which the 41 children in this cohort were treated, we developed the PanTum Detect tests using the EDIM platform as a potential marker of response to treatment [36]. These tests were originally developed for cancer screening purposes [30,31,32,33], but showed value in the temporal monitoring of patient responses [63,64]. Data in GBM patients suggested that high PanTum Detect test scores could reflect responsive patients with better survival [27]. A similar observation was present in this cohort of patients. The kinetics of these markers showed a transient response to the different treatment modalities and allowed a day-by-day assessment of the effect of ICD treatment. Both NDV [41,42] and mEHT [47,48,49,50] have been shown to induce ICD. The in vivo assessment of ICD is difficult, as biomarkers in blood are strongly diluted and are not fully representative of the biological processes occurring within the tumor. Most in vivo assessment of ICD relies on the abscopal effect that can be studied in pre-clinical in vivo models. Intracellular staining of tumor-related markers such as Apo10 and TKTL1 can be considered as a methodology using the circulating CD14+CD16+ myeloid cells in the blood, which may provide information on the tumor. Any kind of tumor cell damage causing leakage of Apo10 and TKTL1 could be picked up by myeloid cells. Thus, the interpretation of the PanTum Detect test scores is complex and influenced by many factors. However, the repetitive day-by-day increase upon ICD treatment might suggest a causal relationship between ICD treatment and increased biomarker levels.

## 5. Conclusions

We report our experiences with multimodal immunotherapy in a large cohort of children with DIPG treated within a short time frame. This retrospective analysis has uncovered several interesting observations that may allow further optimization of multimodal immunotherapy for DIPG as part of primary treatment, focusing on Th1/Th2 shifting, the mode of NDV application, and the intensity of mEHT. The evolution of PanTum Detect test scores may emerge as a tool for assessing treatment response on a daily basis. This retrospective analysis consisted of a biased group of patients, and all associated limitations should be considered. Further preclinical mechanistic data should be generated to support the treatment concept. Nevertheless, the observed median OS of 14.4 months, 1-year survival of 65%, and 2-year survival of 10% for children belonging to intermediate or high-risk profiles remain remarkable. The data suggest that multimodal immunotherapy may be useful when integrated with the first line of treatment. A phase I/II clinical trial incorporating multimodal immunotherapy after the standard radiotherapy, and measuring the variables described here, is the most appropriate design at the current stage. This report aims to provide information to the scientific community for appropriate counseling of patients, and ultimately for consideration of the inclusion of multimodal immunotherapy in innovative clinical trials.
